# Systemic sclerosis-associated myositis features minimal inflammation and characteristic capillary pathology

**DOI:** 10.1007/s00401-021-02305-3

**Published:** 2021-04-17

**Authors:** Elise Siegert, Akinori Uruha, Hans-Hilmar Goebel, Corinna Preuße, Vincent Casteleyn, Felix Kleefeld, Rieke Alten, Gerd R. Burmester, Udo Schneider, Jakob Höppner, Kathrin Hahn, Carsten Dittmayer, Werner Stenzel

**Affiliations:** 1 Department of Rheumatology and Clinical Immunology, Charité-Universitätsmedizin Berlin, Charitéplatz 1, 10117 Berlin, Germany; 2 Berlin Institute of Health, Anna-Louisa-Karsch-Str. 2, 10178 Berlin, Germany; 3 Department of Neuropathology, Charité-Universitätsmedizin Berlin, Corporate Member of Freie Universität Berlin, Humboldt-Universität zu Berlin, and Berlin Institute of Health (BIH), Charitéplatz 1, 10117 Berlin, Germany; 4 Schlosspark-Klinik, Heubnerweg 2, 14059 Berlin, Germany; 5 Department of Neurology and Experimental Neurology, Charité-Universitätsmedizin Berlin, Charitéplatz 1, 10117 Berlin, Germany; 6 Leibniz ScienceCampus Chronic Inflammation, 10117 Berlin, Germany

**Keywords:** Systemic sclerosis, Myositis, Capillary pathology, Large-scale electron microscopy

## Abstract

Systemic sclerosis represents a chronic connective tissue disease featuring fibrosis, vasculopathy and autoimmunity, affecting skin, multiple internal organs, and skeletal muscles. The vasculopathy is considered obliterative, but its pathogenesis is still poorly understood. This may partially be due to limitations of conventional transmission electron microscopy previously being conducted only in single patients. The aim of our study was therefore to precisely characterize immune inflammatory features and capillary morphology of systemic sclerosis patients suffering from muscle weakness. In this study, we identified 18 individuals who underwent muscle biopsy because of muscle weakness and myalgia in a cohort of 367 systemic sclerosis patients. We performed detailed conventional and immunohistochemical analysis and large-scale electron microscopy by digitizing entire sections for in-depth ultrastructural analysis. Muscle biopsies of 12 of these 18 patients (67%) presented minimal features of myositis but clear capillary alteration, which we termed minimal myositis with capillary pathology (MMCP). Our study provides novel findings in systemic sclerosis-associated myositis. First, we identified a characteristic and specific morphological pattern termed MMCP in 67% of the cases, while the other 33% feature alterations characteristic of other overlap syndromes. This is also reflected by a relatively homogeneous clinical picture among MMCP patients. They have milder disease with little muscle weakness and a low prevalence of interstitial lung disease (20%) and diffuse skin involvement (10%) and no cases of either pulmonary arterial hypertension or renal crisis. Second, large-scale electron microscopy, introducing a new level of precision in ultrastructural analysis, revealed a characteristic capillary morphology with basement membrane thickening and reduplications, endothelial activation and pericyte proliferation. We provide open-access pan-and-zoom analysis to our datasets, enabling critical discussion and data mining. We clearly highlight characteristic capillary pathology in skeletal muscles of systemic sclerosis patients.

**Supplementary Information:**

The online version contains supplementary material available at 10.1007/s00401-021-02305-3.

## Introduction

Systemic sclerosis (SSc) is a rare connective tissue disease, characterized by the pathophysiological triad of fibrosis, vasculopathy and autoimmune phenomena in the form of inflammatory cell infiltrates and disease-specific diagnostic antibodies. Almost all patients have anti-nuclear antibodies, and most harbor disease-specific autoantibodies directed against Scl-70, centromeres or RNA polymerase III. SSc can involve sclerosis of the skin and various internal organs potentially leading to terminal organ failure. Raynaud’s phenomenon, a reversible narrowing of peripheral arteries, occurs in most SSc patients. However, there are also structural alterations of vessels: Microscopic evaluation of capillaries at the nailfold typically shows both capillary enlargement and reduced capillary density, and there is well-known obliterative vasculopathy in SSc-associated digital ulcers, pulmonary arterial hypertension (PAH) and scleroderma renal crisis (SRC) [4, 40]. These lesions consist of concentric intimal proliferation, leading to luminal obstruction, and inflammatory cell infiltration [10]. In addition, there are so-called plexiform lesions, which are believed to be caused by hyper-proliferation of endothelial cells in the lung [6].

Light microscopic findings in skeletal muscle of SSc patients were described only in few individuals, consisting of mild alterations including interstitial and perivascular fibrosis, muscle fiber atrophy, inflammatory infiltrates, necrotic muscle fibers and centrally placed myonuclei [7, 21, 32, 39]. Ultrastructural analysis was performed in anecdotal studies, and a small subset of patients revealed only minimal changes and demonstrated breakdown of myofibrils in some atrophic fibers [39], while others [24] described marked changes in numerous myofibers including granular degeneration. Capillaries with thickened walls were also reported, variably caused by thickening and sometimes reduplication of the basement membrane (BM) and endothelial proliferation [24, 32, 39]. However, these studies did not provide an integrated analysis including detailed clinical/laboratory and histological/immunohistochemical analysis. Hence, focal or subtle ultrastructural alterations might have been missed due to limitations of conventional electron microscopy analysis.

Clinically, muscle involvement in SSc is obviously heterogeneous [25]. While there are cases of SSc overlapping with specific idiopathic inflammatory myopathies (IIMs), such as dermatomyositis (DM) or anti-synthetase syndrome in particular [3], there is also a poorly understood muscle disease specific to SSc. This latter is functionally relevant in approximately 20% of all patients [34] and affects both quality of life and disability [16, 26], and has been associated with relevant cardiac complications [12].

This study addresses the specific skeletal muscle phenotype on the light microscopic level with a focus on inflammatory features relevant in myositis [2]. Furthermore, we introduce large-scale electron microscopy (EM) as a new tool for in-depth study of muscle pathology. Here, entire ultrathin sections are automatically digitized at high resolution, and resulting datasets enable efficient pan-and-zoom analysis, annotations and measurements via open-source software [9].

## Materials and methods

### Patient cohort, clinical and laboratory data

Among 367 patients classified as SSc according to the 2013 American College of Rheumatology/European League Against Rheumatism criteria, we identified 18 patients who underwent muscle biopsy due to presence of muscle symptoms. Clinical data were collected in routine clinical practice following standardized European Scleroderma Trials and Research group (EUSTAR) procedures and are summarized in Supplementary Table 1, online resource. All patients were assessed for muscle symptoms (myalgia/muscle weakness) as part of the physical exam, which includes standard neurological examination. Medical research council (MRC) scale-based data were unfortunately not available for all patients. Antibody profiles were determined using EUROLINE ANA profile 3 by Euroimmun for ANA characterization, an ELISA directed against RNA polymerase III by MBL Life Science Corporation and EUROLINE myositis blot by Euroimmun until 2018. For patients assessed afterwards, we used EUROLINE systemic sclerosis profile and EUROLINE autoimmune inflammatory myopathies both by Euroimmun. We performed histopathological analysis according to a standardized protocol in line with the current standard used for the assessment of inflammatory muscle diseases [41] that was slightly adapted. For large-scale digitization, we also included 10 disease controls (8 DM patients’ biopsies and 2 anti-synthetase syndrome patients’ biopsies) who fulfilled the respective ENMC diagnostic criteria [15, 20] and a non-diseased control. Additionally, one non-diseased control was examined by conventional transmission electron microscopy (TEM). Informed consent was obtained from all patients involved. All procedures were approved by the official ethical standards committee (EA2/163/17) at Charité-Universitätsmedizin Berlin.

### Histologic, enzyme histochemical and immunohistochemical procedures

Routine stains were performed on 7 µm cryostat sections according to standard procedures. Immunohistochemical stains were obtained as described previously [28]. The following antibodies were used for staining procedures: C5b-9 (Dako, aE11, 1:200), CD8 (Dako, C8/144B, 1:100), CD20 (Dako, L26, 1:200), CD45 (Dako, 2B11, 1:400), CD56 (Serotec/MCA591 ERIC-1, 1:400), CD68 (Dako, EBM11, 1:100), CD138 (Dako, MI15, 1:30), CD206 (Acris, 7–450, 1:500), ISG15 (abcam, ab14374, 1:100), MHC class I (Dako, w6/32, 1:1000), MHC class II (Dako, CR3/43, 1:100), neonatal MyHC (Novocastra, NB-MHCn, 1:20), MxA (Santa Cruz, sc-50509 polyclonal, 1:100), PDGFR-β (Santa Cruz, sc-339, P-20, 1:30), Siglec1 (Millipore, MABT328 5F1.1, 1:50), SQSTM1/p62 (Abcam, rabbit polyclonal 91526, 1:100). Appropriate positive and negative controls (tissue reactions) were used where necessary. Additionally, normal muscle or physiological control (e.g., staining of arterioles by C5b-9, MHC class I positivity of capillaries) was used as negative control for all reactions as described in Ref. [28]. The above-mentioned comprehensive antibody panel was also used to ensure negative staining results by studying so-called “irrelevant antibodies” for validation.

### Transmission electron microscopy

Muscle specimens were fixed and embedded according to standard protocols. Briefly, muscle specimens were fixed in 2.5% glutaraldehyde in 0.1 M sodium cacodylate buffer for a minimum of 24 h at 4 °C, osmicated in 1% osmium tetroxide in 0.05 M sodium cacodylate buffer, dehydrated using graded acetone series including combined en bloc staining with 1% uranyl acetate and 0.1% phosphotungstic acid in 70% acetone, infiltrated in RenLam resin and then polymerized for 48–72 h at 60 °C. Semithin sections (500 nm) were stained with Richardson solution for microanatomical examination, and ultrathin sections (60–70 nm) were stained with uranyl acetate and lead citrate. Conventional ultrastructural analysis using TEM 902 and TEM 906 (Zeiss, Oberkochen, Germany) was performed only in one non-diseased control case.

### Large-scale digitization (“nanotomy”)

Routinely fixed and embedded muscle samples were used to prepare ultrathin sections on support film-coated slot grids similar as previously described [9, 17, 35]. We digitized one entire ultrathin section per SSc case and disease/pathological control cases and a non-diseased control (*n* = 29). Briefly, a Gemini 300 field-emission scanning electron microscope (FESEM; Zeiss, Oberkochen, Germany), equipped with a scanning transmission electron microscopy (STEM) detector, was used to digitize entire ultrathin sections with Atlas 5 Software (Fibics) at 7.3 nm pixel size and 1 µs dwell time. Using Atlas 5, overlapping image tiles were stitched and exported into an internet browser-compatible format for upload and open-access pan-and-zoom analysis on http://www.nanotomy.org. In addition, image tiles were stitched and exported to bigtif files using Fiji with TrakEM2 plugin [5] (open source software; last accessed 16.09.2020) and nip2 (open source software; https://www.github.com/libvips/nip2/releases, last accessed 16.09.2020) to allow for analysis via QuPath [1] (open source software; https://www.qupath.github.io/, last accessed 16.09.2020).

### Semi-quantitative scoring systems and data analysis

We applied semi-quantitative scores for light microscopic analysis of muscle biopsies using conventional and immunohistochemical stains (see Supplementary Table 3, online resource). Following pseudonymization, whole tissue sections were evaluated by three blinded readers (WS, ES, AU) in a consensus approach. A visual analogue scale (VAS) score for general pathological alteration (severity score) was established with 0 cm = no alterations to 10 cm = most severe alterations [45]. In addition, capillaries, muscle fibers and endomysium/perimysium were evaluated with a score ranging from 0 to 3 that we based on a previously published scoring system [45].

A semi-quantitative score for ultrastructural evaluation of capillary pathology was applied for BM thickening, BM reduplication, endothelial activation, capillary ensheathment by endothelial and/or pericyte processes and presence of tubuloreticular inclusions (TRI) in endothelial cells or pericytes. For this semi-quantitative score, 100 capillaries in each dataset (per patient sample) were manually annotated, except for eight samples containing less than 100 in a section, and afterwards scored by two researchers in a consensus approach (AU, CD) and subsequently discussed with two further authors (WS, HHG). In total, we applied this scoring system to 2618 capillaries (18 SSc cases, 10 disease controls, 1 non-diseased control). 0 = 50–100 nm BM thickness, no reduplication, no endothelial activation, no prominent ensheathment (1–4 processes), no TRI; 1 = 100–200 nm BM thickness, mild reduplication (2–3 layers), mild endothelial activation (increased area and/or organelles), prominent ensheathment (5–6 processes, not only focal and small), 2 small- or 1 medium-sized TRI; 2 = 200 + nm BM thickness, marked reduplication (4 + layers), marked endothelial activation, very prominent ensheathment (7 + processes, also depending on size), 2 + medium- or 1 + large-sized TRI.

Data analysis was performed with Excel. An average sum (AS) score was generated to assess general capillary pathology per case by adding individual capillary scores of the categories: BM thickening, BM reduplication, endothelial activation and capillary ensheathment and dividing by the analyzed capillaries. In addition, an average category sum (ACS) score was generated to assess the portion of each of these four categories per case. Beside scoring of capillaries, all datasets of SSc cases were also examined in detail via QuPath to evaluate ultrastructural findings in addition to the described capillary pathology.

The cases were sorted by the VAS-score and the AS-score for visualization of the EM scoring data, together with age and creatinine kinase (CK) levels, light microscopic findings and additional EM findings. Micrographs were edited using Adobe Photoshop CC, and figures were prepared with CorelDRAW 2020. Graphs of scoring data were prepared with Prism GraphPad 9.0.0.

## Results

### Clinical aspects

**Fig. 1:**
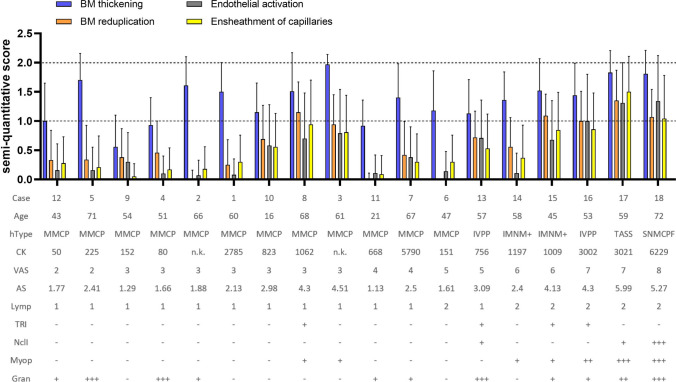
Representation of capillary scoring analysis of all systemic sclerosis (SSc) patients, visualized with age and CK levels and based on light microscopy and large-scale digitization datasets, also showing additional ultrastructural findings. Average category sum (ACS) scores of basement membrane (BM) thickening (blue), BM re-duplication (orange), endothelial activation (gray) and ensheathment of capillaries by pericyte and/or endothelial processes (yellow). All 12 SSc cases with a histological pattern (hType) of minimal myositis with capillary pathology (MMCP) show low to medium visual analogue scale (VAS) score values. Here, cases 10, 8 and 3 demonstrate highest average sum (AS) and ACS scores and also presence of myophagocytoses (Myop; 8, 3) and tubuloreticular inclusions (TRI; 8), while all other MMCP cases only show mild capillary alterations. Note that all cases with non-MMCP histological pattern (13–18) demonstrate high VAS scores and also high AS and ACS scores, while also showing presence of myophagocytoses (14–18), TRI (13, 15, 16) and nuclear inclusions (13, 17, 18). Abbreviations: CK creatine kinase, Lymp lymphocyte infiltrates, Gran granular degeneration of myofibers, IVPP inflammatory vasculopathy with perimysial pathology, IMNM + immune-mediated necrotizing myopathy with MHC-II positivity, TASS typical anti-synthetase syndrome, SNMCPF severe necrotizing myositis with capillary pathology and fibrosis. Displayed are bar graphs with mean and SD

All patients included in our study fulfilled the ACR/EULAR 2013 classification criteria for SSc (Fig. 1; Supplementary Table 1, online resource). Briefly, all patients were ANA-positive, but only half of them had SSc-specific antibodies, namely five patients with anti-centromere antibodies (ACA), two with anti-Scl-70 antibodies and two with anti-polymerase III (Poly III) antibodies. There were more females than males in our cohort (72%/28%), and at the time of biopsy mean age was 54 years, and mean disease duration was three years. Limited cutaneous SSc (lcSSc) was present in 75% of all patients, while 25% had diffuse cutaneous SSc (dcSSc); this is in line with the reported distribution of cutaneous involvement in large European SSc cohorts [22]. Concerning other organ involvement, there was a prevalence of 38% of interstitial lung disease (ILD) and 6% of PAH. Cardiac involvement was present in 31% of all patients, while 38% had a history of digital ulcers. There was no patient with a history of SRC. The subgroup of patients receiving a muscle biopsy had relatively early and mild disease except for a high prevalence of cardiac involvement compared to the non-biopsied cohort (for details see Supplementary Table 1, online resource). Mean maximum serum CK levels were on average tenfold elevated at 1752 (± 1923) U/l (reference value CK for males < 190 U/l; for females < 167 U/l).

### Light microscopic analysis reveals a unique pattern of systemic sclerosis-associated myositis: minimal myositis with capillary pathology; MMCP

**Fig. 2:**
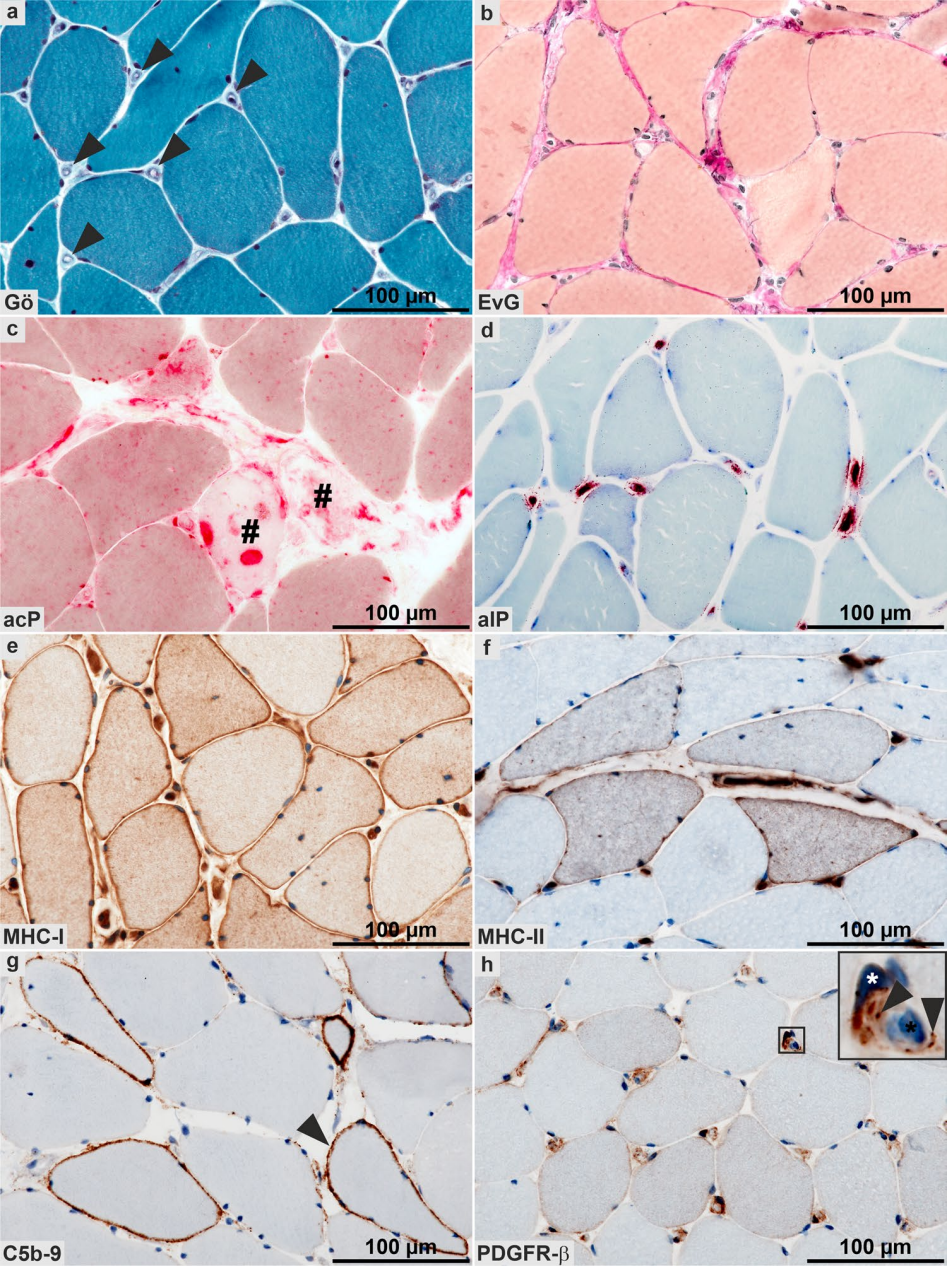
Light microscopic features of skeletal muscle biopsies with histological minimal myositis with capillary pathology (MMCP) phenotype. Representative micrographs of patient 8. **a** Markedly thickened endomysial capillaries (arrowheads) in the Gömöri trichrome (Gö) stain and mild endomysial fibrosis in the Elastica-van-Gieson (EvG; **b**) stain. Myophagocytoses (#) are highlighted in the acid phosphatase (acP; **c**) reaction, and alkaline phosphatase (alP; **d**) is demonstrated in multiple endomysial capillaries. Immunohistochemistry shows mild sarcoplasmic and sarcolemmal staining of major histocompatibility complex class I (MHC-I; **e**) in numerous fibers, and also of major histocompatibility complex class II (MHC-II; **f**) in several fibers. **g** Focal C5b-9 complement deposition on the sarcolemma (arrowhead). **h** Platelet-derived growth factor receptor beta (PDGFR-β) highlights prominent pericytes around capillaries: note that some capillaries demonstrate distinct ensheathment by multiple pericyte processes, however, due to limited resolution of conventional light microscopy these cannot clearly be differentiated (inset: endothelial cell nucleus, black asterisk; pericyte with nucleus and large process, white asterisk; multiple small pericyte processes, arrowheads)

12/18 biopsies revealed strikingly similar histopathological features that we termed “minimal myositis with capillary pathology” (MMCP; Fig. 1, 2; Supplementary Tables 2–4, online resource). This newly identified entity is characterized as a mild form of myositis with sparse endomysial T-lymphocytic infiltrates and absence of any muscle fiber invasion. Necrotic fibers were exceptional, no relevant myophagocytosis and occasional regenerating fibers occurred. There were almost no B cells noted, and relatively little endomysial and perimysial macrophages. Immunohistochemistry for type I interferon-induced proteins, MxA and ISG15, on the sarcoplasm and capillaries, was negative. MHC class I was variably detected on the sarcolemma of many fibers with a diffuse distribution, while MHC class II was only detected on sparse myofibers (Fig. 2e, f), and rarely, focal complement deposition on the sarcolemma but not on capillaries was observed (Fig. 2g). Of note, the most striking finding in most cases, was that many enlarged capillaries harbored prominent PDGFR-β-positive pericytes (Fig. 2h) and some also showed ensheathment by multiple layers of pericyte processes.

6/18 skeletal muscle biopsies demonstrated different, non-MMCP histological patterns (Fig. 1; Supplementary Table 2, online resource), including one case with typical features of anti-synthetase syndrome-associated myositis [23, 36, 44] and five cases that were not classifiable according to prevailing classification schemes, and therefore were termed as non-specific myositis or clinically as overlap myositis [2, 15]. All of these six skeletal muscle biopsy specimens demonstrated striking differences as compared to the MMCP phenotype, showing a prominent sarcolemmal MHC class I, and of note, also MHC class II positivity, as well as prominent endomysial and perimysial inflammatory infiltrates and necrotic myofibers. These cases also harbored thickened enlarged capillaries on the light microscopic level and had higher VAS scores as compared to MMCP cases (Fig. 1).

Interestingly also, some of the non-MMCP cases showed distinct histopathological features associated with other pathologies/entities. There was one case with an anti-synthetase pattern on biopsy, one case of a severe necrotizing myositis with capillary pathology and fibrosis and two patients each with an ‘immune-mediated necrotizing’-like myopathy but with additional inflammatory vasculopathy with perimysial pathology. The patient with an anti-synthetase pattern on biopsy was positive for both the anti-synthetase antibody PL7 and the SSc antibodies ACA and Poly III.

From a clinical point of view, MMCP cases were characterized by a high percentage of lcSSc (90%), and relatively mild disease with very few patients presenting with ILD (20%) or other organ involvement (40% with a history of digital ulcers, 20% with cardiac involvement, no PAH or SRC). Also, maximum CK levels were lower with a mean value of 1325 (± 1791) U/l while the non-specific cases had a mean value of 2536 (± 2068) U/l. Interestingly, the non-specific cases had more severe disease with a higher percentage of patients presenting with dcSSc (50%), ILD (67%) and cardiac involvement (50%). However, these differences missed statistical significance. Non-SSc cases (disease control muscle specimens) with anti-TIF1γ, -Mi-2, -NXP2 and -MDA5-positive DM, and also anti-Jo-1-associated anti-synthetase syndrome showed the characteristic light-microscopic features described in detail recently [2, 38]

### Ultrastructural analysis reveals a distinct set of capillary pathology

**Fig. 3:**
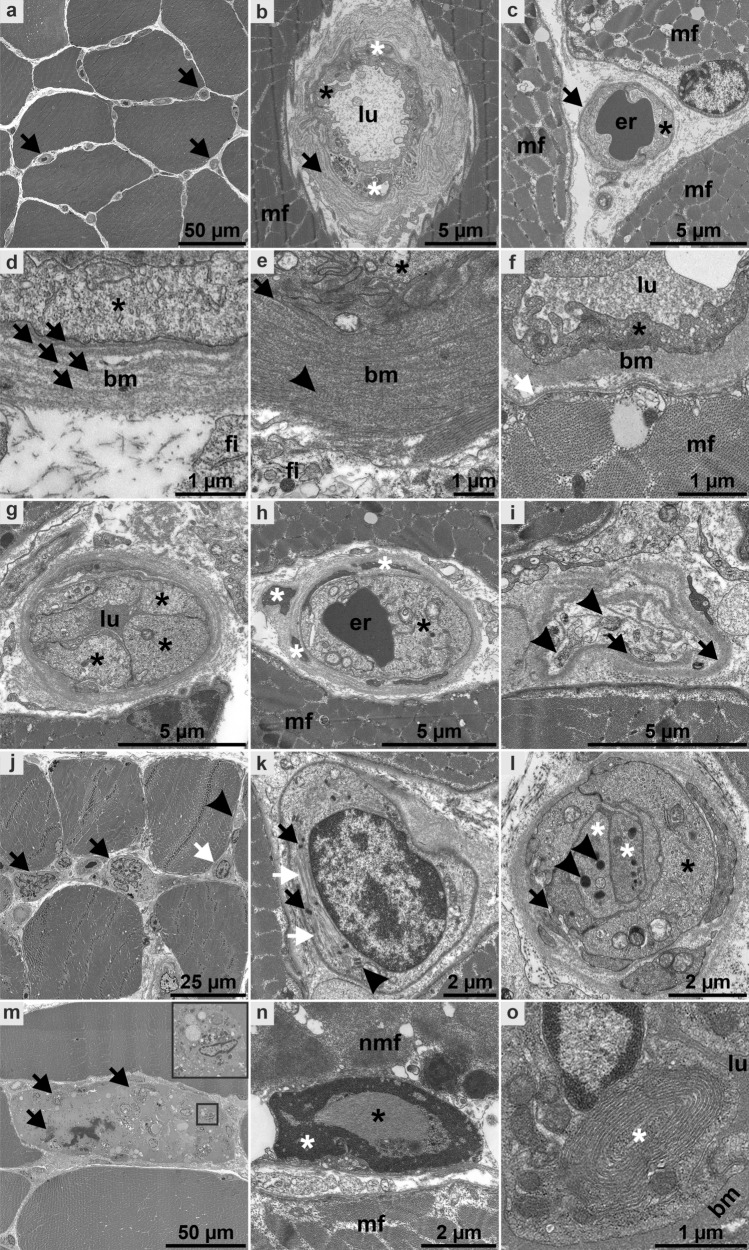
Ultrastructural characterization of skeletal muscle biopsies with histological minimal myositis with capillary pathology (MMCP) phenotype. Entire ultrathin sections were recorded by large-scale digitization at 7.3 nm pixel size; digitally magnified regions of interests (ROI) of patients 3 (**a**, **d**, **g**–**l**), 8 (**b**, **m**), 6 (**e**), 2 (**f**), 18 (**n**; severe necrotizing myositis with capillary pathology and fibrosis), 1 (**o**) and non-diseased control (**c**). **a** Markedly thickened capillaries can be clearly identified at low magnification (arrows). **b** Capillary with pronounced thickening and reduplication (arrow) of the basement membrane (BM) as well as ensheathment by pericyte processes (white asterisk), mildly activated endothelium (black asterisk), lumen (lu), adjacent muscle fiber (mf). **c** Healthy capillary with thin BM (arrow): note that no reduplication is apparent, endothelium with no signs of activation (asterisk), lumen with erythrocyte (er). Different types of BM (bm) thickening were detected (**d**–**f**): Distinct reduplication (**d**; arrows), endothelium (asterisk), fibroblast (fi); fuzzy appearance with less pronounced reduplication (**e**): note that the BM (arrow) directly underneath the endothelium (asterisk) is clearly visible, fibrous long spacing collagen (arrowhead); homogeneous thickened BM (**f**), capillary lumen (lu), neighboring muscle fiber (mf) with basal lamina (white arrow). Different types of endothelial activation (**g**–**i**): Increased size and number of endothelial cells (**g**; asterisk, lu lumen), note the granular appearance of the cytoplasm, probably mostly due to ribosomes; prominent endothelial membrane organelles (**h**; black asterisk, er erythrocyte in lumen), note the prominent and mostly small pericyte processes demonstrating mild ensheathment (white asterisks); degraded capillary (**i**), showing some remaining membrane structures, probably of endothelial or pericyte origin (arrowheads), almost empty BM “sack” with mild reduplication (arrows). Examples of additional findings in the examined large-scale datasets (**j**–**o**): **j** numerous atrophic muscle fibers were observed in patient 3 (white arrow), including multiple nuclear clumps (black arrows), location of the capillary remnant shown in **i** (arrowhead). **k** Atrophic muscle fiber shown in **j**, digitally magnified, demonstrating IZI-bands (arrowhead), clumps of z-band material (black arrows) and some remaining filaments (white arrows). **l** Capillary with markedly activated endothelium (black asterisk), the lumen seems to be occluded by two thrombocytes (white asterisks; two dense core granules, arrowheads), note the small endothelial process (arrow). **m** Myophagocytosis; macrophages with electron dense material (arrows, also inset). **n** Nuclear filamentous inclusions were observed in some non-MMCP patients (black asterisk; heterochromatin, white asterisk), note a presumable necrotic muscle fiber (nmf) compared to normal muscle fiber (mf). **o** In single endothelial cells, distinct membrane structures of lamellated character (asterisk) were found, BM (bm) and lumen (lu)

Based on digitization of entire ultrathin sections of 18 SSc (MMCP versus non-MMCP) cases and 11 control cases (10 disease controls and 1 non-disease control), we analyzed 2618 capillaries by our visual scoring system. We focused on BM thickening, reduplication, endothelial activation, capillary ensheathment by endothelial and/or pericyte processes, presence of TRI, and also additional (non-vessel related) findings (Figs. 1, 3; Supplementary Fig. 1, Supplementary Tables 5, 6, online resource).

The 12 analyzed MMCP-specimens demonstrated lower AS scores (between 1.13 and 4.51; 2.35 ± 1.09) as compared to the non-MMCP-specimen (between 2.40 and 5.99; 4.2 ± 1.33). Interestingly, the MMCP-specimens 3, 8 and 10 showed relatively high AS scores, caused by relatively high ACS scores of BM reduplication, endothelial activation and ensheathment. Other MMCP-specimens showed either mild (1, 4, 5, 7, 9) or very mild (2, 6, 11) alterations of these three categories. Specimens 3, 8 and 10 were also characterized by presence of TRI (8) and myophagocytosis (3, 8), while the other MMCP specimens did not show either of these structures. Contrarily, among the non-MMCP-specimens, presence of TRI and myophagocytosis were strikingly frequent features, and also myonuclear inclusions were found in three specimens.

These features were absent in a digitized ultrathin section of the non-disease skeletal muscle biopsy (AS score 0.08; Supplementary Fig. 1, online resource). Disease control muscle specimens belonging to anti-TIF1γ, -Mi-2, -NXP2 and -MDA5-positive DM, and also anti-Jo-1-associated anti-synthetase syndrome revealed a strikingly different aspect of capillary pathology (Supplementary Fig. 1, online resource). DM specimens generally showed lower AS scores (between 0.36 and 2.56; 1.45 ± 0.68), similarly did both anti-synthetase syndrome specimens (2.56 and 4.55). Of note, the DM specimens revealed overtly different AS and ACS scores, depending on their antibody subtypes (Supplementary Fig. 1, online resource).

## Discussion

In this study, we precisely characterized muscle pathology in SSc using histological, enzyme histochemical and immunohistochemical procedures in conjunction with an innovative EM technology that allows unrestricted in-depth analysis of entirely digitized ultrathin sections. We integrated these results with detailed clinical and serological data to further specify the relevance of our findings and to gain new pathogenic insights into the disease. Specifically, we looked for unique features putatively distinguishing SSc-associated muscle disease from other IIMs, as SSc-associated muscle disease is not considered part of the IIM ‘superfamily’. We were able to delineate a homogeneous group that we termed MMCP phenotype. MMCP features striking homogeneous morphological patterns as compared to the different non-MMCP cases and is recognizably characterized by mild myositis attributes, associated with capillary pathology on the light microscopic level. Here, ultrastructural examination including semi-quantitative analysis of 2618 capillaries allowed to precisely assess distinct subtypes within the specimens that demonstrated an MMCP phenotype. We identified capillary pathology in all skeletal muscle biopsies, however, the intensity and quality of alterations varied considerably between the MMCP and in the non-MMCP group. In all cases, we found capillary alterations characterized by thickening of BM, reduplication of BM, endothelial activation and ensheathment by endothelial and pericyte processes to different degrees obviously representing different stages of the capillary affection. The latter were milder in MMCP cases compared to non-MMCP cases; non-MMCP cases also showed additional alterations, such as nuclear inclusions, myophagocytosis and higher VAS score. Of note, MMCP features in SSc-associated myositis patients were reflected by clinically mild disease with a high percentage of lcSSc, few internal organ involvements and low maximum CK levels.

Pericytes are known to guide angiogenesis of vessels, and abnormal features of these cells are known to occur in patients with early SSc [31] and have been suggested as a link between vasculopathy and fibrosis [30]. Here, we were able to highlight their presence and abundance on light- and electron microscopical levels. Enlargement of capillaries was the key finding in all SSc patients’ biopsies; however, we found them in MMCP cases even without prominent inflammatory damage, and this prompted us to precisely study their character. At variance with what is known about capillary pathology, e.g. in DM, no perifascicular atrophy or any perifascicular pathology as in DM or anti-synthetase syndrome [2] was noted. Conversely, atrophic but either no or only single necrotic myofibers were diffusely distributed in the biopsies (Figs. 1, 2c, 3m). These features were absent in non-diseased skeletal muscle. Disease controls, consisting of anti-TIF1γ, -Mi-2, -NXP2 and -MDA5-positive DM muscle specimens, showed capillary pathology with TRI and reduced capillary density, as well as perifascicular muscle fiber atrophy. This intrinsically heterogeneous group of DM is typically characterized by a specific type of vascular pathology and immune-inflammatory features [14, 18, 20, 42, 43], thus distinguishing them clearly from SSc with MMCP (Fig. 1; Supplementary Fig. 1, online resource). These DM features have been previously described [11, 13, 19, 29], and there is a hypothesis linking the prevailing type I interferon signature [33, 37] with DM-vasculopathy pathogenically [18]. In line with this, nuclear inclusions that were found in the three non-MMCP specimens were identified in anti-synthetase syndrome-associated myositis previously [36]. These ultrastructural features clearly differentiate MMCP from non-MMCP specimens and, taking clinical aspects into account, open up the hypothesis that specimens from non-MMCP patients probably fall into a different ‘typical’ overlap subgroup. Hence, we show here that this subgroup is clearly distinguishable from MMCP on morphological grounds.

Innovative EM technology enabled digitization of entire ultrathin sections, thus allowing to preserve ultrastructural details as parts of the microanatomical overview in form of coherent datasets. These datasets allow efficient pan-and-zoom analysis based on conventional personal computers and manual assessment and annotation of, e.g., capillaries for consecutive analysis and discussion in the larger community using open-source software. Hence, this technique clearly surpasses conventional TEM analysis that is based on pre-selected overview and detail images, complicating orientation and efficient analysis of samples that might otherwise require several hundreds of such micrographs per sample. However, the new character of this technique that entails a multitude of ultrastructural information also requires new approaches for analysis as shown in our work. We offer open access to these datasets for online pan-and-zoom examination via an accessible repository (http://www.nanotomy.org) to allow a critical discussion regarding the character of this technique in general, and also for specific morphological interpretation and data mining [8]. We also provide bigtif files on request to include our cases for in-depth analysis in further studies. We think that even more sophisticated tools, such as machine learning, could allow a refined categorization of morphological characteristics.

Serologically, SSc patients with an MMCP pattern had only mild maximum CK elevation with an average of 1325 U/l (± 1791). Concerning autoantibody profiles 4/10 patients had anti-centromere antibodies, which are typically associated with a mild SSc phenotype, while there was one patient each with anti-Ku, anti-Scl-70 and anti-RNA polymerase III antibodies (for details see Supplementary Table 4, online resource). Of note, one patient, positive for anti-RNA polymerase III antibodies, showed severe muscle fiber atrophy. Interestingly, there was another patient positive from the non-MMCP group, harboring amongst others, anti-RNA polymerase III antibodies, who also showed signs of severe muscle fiber atrophy. However, this latter patient had an overall muscle pathology typical for anti-synthetase syndrome which includes severe atrophy, but also various other distinct morphological changes [2]. In accordance with the histopathological findings, this patient was positive for anti-PL7 antibodies, which is the ‘dominant’ autoantibody of several ones in this patient. Thus, the diverse autoantibody profiles found in SSc patients might provide an explanation for the diversity of clinical and morphological features of muscle disease of the remaining 33% SSc patients with a non-MMCP pattern. The presence of immunosuppressive therapy, arterial hypertension or diabetes mellitus at the time of biopsy did not influence the specific MMCP pattern (for details see Supplementary Table 4, online resource). Of note, we did not observe a clear distinction between fibrosing and non-fibrosing muscle disease as described by Paik et al. [27], but rather a continuum of mild fibrosis in all MMCP cases and one case of severe fibrosis in a patient with severe necrotizing myositis and capillary pathology.

As a retrospective study, this study has several limitations. First, all clinical data were collected in routine practice by different clinicians; hence, assessments might be prone to bias. Also, data on muscle weakness were not quantified in all patients; hence, there was no detailed information on functional status and muscle strength associated with the different pathologies. Lastly, there was no systematic algorithm according to which patients received a muscle biopsy or not. Therefore, our biopsied cohort is not entirely representative for SSc patients with muscle disease.

In sum, we integrated clinical, serological and morphological data and show that MMCP is a characteristic and unique pathological pattern in SSc-associated muscle disease. Importantly, pericytes might be critical in the pathogenesis of this specific pathology. From a clinical perspective, it is striking that the extensive findings on muscle biopsies of the 12 MMCP cases were present even with a short mean disease duration at the time of biopsy of three years and with 6/12 cases having a disease duration of less than one year. Also, as compared to the rest of our cohort, patients with MMCP findings on muscle biopsy had a remarkably mild disease with low prevalence of dcSSc and of severe internal organ involvement and low maximum CK levels (for details see Supplementary Tables 1 and 4, online resource). In both groups, MMCP and non-MMCP, there were myositis-specific antibodies present either with or without the presence of SSc-specific antibodies, which are not associated with a specific morphological pattern.

## Supplementary Information

Below is the link to the electronic supplementary material.

Supplementary file1 (PDF 555 KB)
