# Supplementary material for: Systemic sclerosis-associated myositis features minimal inflammation and characteristic capillary pathology
Source: Acta Neuropathol. 2021 Apr 17;141(6):917–27. doi: 10.1007/s00401-021-02305-3 (PMC8113184; doi:10.1007/s00401-021-02305-3)
Supplement: Supplementary file 1 — Supplementary file1 (PDF 555 KB) [file 401_2021_2305_MOESM1_ESM.pdf]

Supplementary Information for

Systemic sclerosis-associated myositis features minimal inflammation and characteristic capillary pathology

Elise Siegert[1,2#], Akinori Uruha[3#], Hans-Hilmar Goebel[3], Corinna Preuße[3], Vincent Casteleyn[1], Felix Kleefeld[5], Rieke Alten[4], Gerd R. Burmester[1], Udo Schneider[1], Jakob Höppner[1], Kathrin Hahn[5], Carsten Dittmayer[3§], Werner Stenzel[3,6§*]

[#] These authors contributed equally

[§] These authors contributed equally

[*] **Corresponding author:**

Prof. Dr. med. Werner Stenzel

Department of Neuropathology

Charité – Universitätsmedizin Berlin, Charitéplatz 1

10117 Berlin, Germany

werner.stenzel@charite.de

telephone number: +49/ 30 450 536 073

fax number: +49/ 30 450 536 940

Supplementary Table 1. Clinical characteristics of all patients with systemic sclerosis

Parameter	biopsy group	non-biopsy group	p-value
Patients (n=)	18	349	
Age (years)	53.8 ± 15.4	53.4 ± 14.0	0.616
Female	72.2%	84.5%	0.166
Disease duration (years)	3.0 ± 5.5	5.7 ± 6.5	**0.001**
Anti-nuclear antibody (ANA)	100%	99.7%	0.829
Anti-Scl70 antibody (anti-topoisomerase 1-antibody)	12.5%	46.3%	**0.008**
Anti-centromere antibody	31.3%	42.7%	0.367
Anti-RNA polymerase III antibody	12.5%	10.3%	0.788
Raynaud symptoms	100.0%	99.7%	0.830
Limited cutaneous systemic sclerosis (lcSSc)	75.0%	62.6%	0.315
Diffuse cutaneous systemic sclerosis (dcSSc)	25.0%	37.4%	0.315
Sine scleroderma	0%	4.0%	0.412
Interstitial lung disease (ILD)	37.5%	2.3%	0.315
Pulmonal-arterial hypertension (PAH)	6.3%	34.8%	0.824
Scleroderma renal crisis (SRC)	0%	7.5%	0.859
Digital ulcers	37.5%	4.0%	0.412
Cardiac involvement	31.3%	16.7%	0.050
Muscle weakness	100%	47.7%	0.000

Data are presented as mean +/- standard deviation; disease duration refers to the time since the first non-Raynaud symptom. Statistical significance was assessed using the Mann-Whitney U test for continuous variables and the Pearson Chi-Square test for categorial variables.

Supplementary Table 2. Histomorphological findings among systemic sclerosis patients according to defined histological subgroup (descriptive nomenclature)

	**MMCP**	**Anti-synthetase**	**IMNM+**	**Severe necrotizing myositis with CP and fibrosis**	**Inflammatory vasculopathy with CP**
n	12	1	2	1	2
VAS	3.17 ± 0.83	7	6.00 ± 0.00	8	6 ± 1.41
Regeneration	1.00 ± 0.60	3	1.50 ± 0.71	3	2 ± 0.71
Fragmentation (perimysium)	0.42 ± 0.51	2	0.00 ± 0.00	2	1 ± 0.00
Fiber atrophy	1.33 ± 0.65	3	2.00 ± 0.00	3	2 ± 0.71
Fibrosis (endomysium)	0.58 ± 0.67	1	0.00 ± 0.00	3	1 ± 0.00
Necrosis	0.75 ± 0.62	3	1.50 ± 0.71	3	2 ± 0.71
Capillary enlargement	1.50 ± 0.80	2	2.00 ± 1.41	3	1 ± 0.00
Reduced capillary density	1.33 ± 0.49	2	1.00 ± 0.00	2	1 ± 0.00
IFN I sig. (ISG15, MxA)	0.00 ± 0.00	0	0.50 ± 0.71	0	0 ± 0.00
Lymphocytes	1.08 ± 0.29	2	2.00 ± 0.00	2	2 ± 0.71
Macrophages (CD68)	1.33 ± 0.49	3	2.00 ± 0.00	3	2 ± 0.71
MHC I (sarcolemmal)	1.25 ± 0.45	2	3.00 ± 0.00	2	3 ± 0.71
MHC II (sarcolemmal)	0.58 ± 0.67	2	2.00 ± 0.00	2	2 ± 0.00
CD56 (turnover)	1.25 ± 0.62	2	1.50 ± 0.71	2	1 ± 0.71
MHCn	0.90 ± 0.88	2	1.50 ± 0.71	3	1 ± 0.00
C5b-9	1.00 ± 0.74	1	1.00 ± 0.00	1	1 ± 0.71

Abbreviations: CD cluster of differentiation, CP capillary pathology, C5b-9 C5 and formation of complement membrane attack complex, IFN I sig. type I interferon signature, IMNM+ immune-mediated necrotizing myopathy with MHC II positivity, ISG15 interferon-stimulated gene 15, MHCn myosin heavy chain neonatal, MHC I major histocompatibility complex class I expression, MHC II major histocompatibility complex class II expression, MMCP minimal myositis with capillary pathology, MxA myxovirus resistance protein 1 on muscle fibres, VAS visual analogue scale. Scales: VAS ranges from 0-10 for severity of muscle damage where 0=no damage and 10=severe damage; all other histomorphological changes are rated from 0-3 where 0=not present and 3=high presence.

Supplementary Table 3. Scoring system for light microscopic analysis (a) and histomorphological data of all muscle biopsies presenting with MMCP pattern (b)

(a)

Scoring category	0	1	2	3
Muscle fiber regeneration (H&E, Gömöri)	absent	few	moderate	intense
Fragmentation of perimysium (EvG)	absent	few/mild	moderate	intense
Atrophy of muscle fibers (H&E, Gömöri)	absent	few/mild	moderate	intense
Endomysial fibrosis (EvG)	absent	few/mild	moderate	intense
Muscle fiber necrosis (H&E, Gömöri)	absent	few/mild	moderate	intense
Capillary enlargement (H&E, Gömöri)	absent	few/mild	moderate	intense
Reduced capillary density (MHC class II)	regular	mildly reduced	moderately reduced	markedly reduced
IFN 1 signature/ MxA and ISG15 staining on fibers	absent	mild	moderate	intense
Endomysial/perimysial lymphocytes (CD8)	absent	few <15/10 HPF	moderate 15-50/10 HPF	intense >50/10 HPF
CD68-positive macrophages	absent	few <30/10 HPF	moderate 30-100/10 HPF	intense >100/10 HPF
Sarcolemmal MHC class I and II staining intensity	no staining	focal staining of < 30% of myofibers	30% - 60% of myofibers (and/or focal accumulation)	> 60% of myofibers
CD56 positive fibers as marker for regeneration	absent	few/mild <5%	some/moderate 5-15%/	numerous/intense >15%/
MHCn positive fibers as marker for regeneration	absent	few/mild <1%	some/moderate 1-5%	numerous/intense >5%
Capillary C5b-9 deposition	absent	few/mild <5%	multiple/moderate 5-15%	numerous/intense >15%

(b)

	VAS	Reg.	Fragmentation (perimysium)	Fiber atrophy	Fibrosis (endomysium)	Necrosis	Cap. enlargement	Reduced cap. density	IFN I sig. (ISG15, MxA)	Lymphocytes	Macrophages (CD68)	MHC I (SL)	MHC II (SL)	CD56 (turn-over)	MHCn	C5b-9 (SL, a/o cap.)
Patient 1	3	1	1	1	1	1	1	1	0	1	1	1	0	1	1	2
Patient 2	3	0	0	1	0	1	1	1	0	1	1	1	0	1	0	1
Patient 3	3	1	1	1	0	1	2	1	0	1	1	1	1	1	1	1
Patient 4	3	0	0	1	0	0	2	2	0	1	2	2	2	1	0	0
Patient 5	2	1	0	1	0	0	2	2	0	1	1	1	0	1	1	0
Patient 6	5	2	0	3	2	1	2	2	0	2	2	2	0	2	3	1
Patient 7	4	1	0	2	0	1	2	2	0	1	2	1	0	3	1	1
Patient 8	3	1	0	2	1	2	3	1	0	1	1	2	1	1	1	2
Patient 9	3	1	1	1	0	0	0	1	0	1	1	1	1	1	0	1
Patient 10	3	1	1	1	1	1	1	1	0	1	1	1	1	1	1	2
Patient 11	4	2	1	1	1	1	1	1	0	1	2	1	1	1	1	1
Patient 12	2	1	0	1	1	0	1	1	0	1	1	1	0	1	0	0

Abbreviations: a/o and/or, cap. capillary, CD cluster of differentiation, C5b-9 C5 and formation of complement membrane attack complex, EvG Elastica van Gieson stain, HPF high-power field, IFN I sig. type I interferon signature, ISG15 interferon-stimulated gene 15, LC sarcolemmal, MHCn myosin heavy chain neonatal. MHC I major histocompatibility complex class I expression, MHC II major histocompatibility complex class II expression, MxA myxovirus resistance protein 1 on muscle fibres, Reg. regeneration, VAS visual analogue scale. Scales: VAS ranges from 0-10 for severity of muscle damage where 0=no damage and 10=severe damage; all other histomorphological changes are rated from 0-3 where 0=not present and 3=high presence.

Supplementary Table 4. Clinical data of all patients presenting with MMCP pattern on muscle biopsy

	Female	Disease duration (years)	Age at biopsy (years)	Antibodies	Highest CK value (U/l)	Cutaneous involvement	Organ involvement	Immunosuppressive treatment at biopsy	Arterial hypertension	Diabetes mellitus type II
Patient 1	y	0	60	Ku, RF IgM	2785	lcSSc	heart, digital ulcers	n	n	n
Patient 2	y	n.k. (> 0)	66	n.k.	n.k.	n.k.	n.k.	n.k.	n.k.	n.k.
Patient 3	n	0	61	n.k.	n.k.	n.k.	n.k.	n.k.	n.k.	n.k.
Patient 4*	y	0	51	ACA	80	lcSSc		n	y	n
Patient 5*	y	20	71	ACA, U1RNP	225	lcSSc		azathioprine	y	n
Patient 6*	y	6	47	RP3, AT1R, ETAR	151	lcSSc	ILD	n	n	n
Patient 7	y	11	67	ACA	5790	lcSSc	digital ulcers	hydroxychloroquine	n	n
Patient 8	n	0	68		1062	lcSSc	heart, digital ulcers	n	y	n
Patient 9	n	0	54		152	lcSSc		n	n	n
Patient 10	y	0	16	Scl-70	823	dcSSc	ILD	n	n	n
Patient 11	y	1	21	U1RNP	668	lcSSc		n	n	n
Patient 12	y	4	43	ACA, PM-Scl, SAE1, Ro52, cANCA, RF IgM, AMA M2, ACPA	50	lcSSc	digital ulcers	hydroxychloroquine	n	n

Abbreviations: ACA Anti-centromere antibody, ACPA anti-citrullinated protein antibody, AMA M2 anti-mitochondrial antibody M2 subtype, AT1R anti-angiotensin I Receptor antibody, cANCA cytoplasmatic antineutrophil cytoplasmic autoantibodies, ETAB anti-Endothelin Receptor A Receptor antibody, dcSSc diffuse cutaneous Systemic Sclerosis, ILD Interstitial lung disease, Ku anti-Ku antibody, lcSSc limited cutaneous Systemic Sclerosis, n no, n.k. not known, PM-Scl anti-PM-Scl antibody, RF Rheumatoid factor, Ro52 anti-Ro52 antibody, RP3 anti-RNA polymerase III antibody, SAE1 anti-SAE1 antibody, Scl-70 anti-Scl 70 antibody, U1RNP anti-U1-RNP antibody, VAS visual analogue scale, y yes.

Note that there were three cases of malignancy (namely breast, colon and lung cancer; patients marked by *) among the 12 patients classified with MMCP with two being positive for anti-centromere antibodies and one positive for RNA polymerase III antibodies. While patients with anti-RNA polymerase III antibodies are known to be at risk for malignancies, anti-centromere antibodies are thought to be protective [13].

Supplementary Table 5. Additional electron microscopical findings and information of all 18 systemic scleosis cases

	VAS	Histo	AS	Capillary remnants	TRI	Fibrosis	Infiltrates	Atrophic fibers	Centrally placed myonuclei	Nuclear inclusions myofibers	Myophago-cytosis	Granular material myofibers
Patient 1	3	MMCP	2.13	-	-	+	+	-	+	-	-	-
Patient 2	3	MMCP	1.88	-	-	-	-	-	-	-	-	+
Patient 3	3	MMCP	4.51	+++	-	+	++	+++	+	-	+	-
Patient 4	3	MMCP	1.66	-	-	-	+	-	-	-	-	+++
Patient 5	2	MMCP	2.41	-	-	-	-	++	-	-	-	+++
Patient 6*	5	MMCP	1.61	-	-	++	-	+++	-	-	-	-
Patient 7	4	MMCP	2.5	+	-	+	-	+++	+	-	-	+
Patient 8	3	MMCP	4.3	+	+	+	+	-	+	-	+	-
Patient 9	3	MMCP	1.29	+	-	+	+	+	++	-	-	-
Patient 10	3	MMCP	2.98	+	-	+	+	-	-	-	-	-
Patient 11	4	MMCP	1.13	-	-	+	+	-	-	-	-	+
Patient 12	2	MMCP	1.77	+	-	+	-	-	-	-	-	+
Patient 13	5	IVPP	3.09	-	+	-	++	+	+++	+	-	+++
Patient 14	6	IMNM+	2.4	-	-	-	+	+	+	-	+	-
Patient 15	6	IMNM+	4.13	+	+	-	++	++	+	-	+	+
Patient 16	7	IVPP	4.3	+	+	+	+	++	-	-	++	+
Patient 17	7	TASS	5.99	-	-	++	+	+	++	+	+++	++
Patient 18	8	SNMCPF	5.27	+	-	-	++	++	+	+++	+++	+++

Abbreviations: AS average sum score, Histo histological diagnosis, IMNM+ immune-mediated necrotizing myopathy with MHC II positivity, IVPP inflammatory vasculopathy with perimysial pathology, MMCP minimal myositis with capillary pathology, SNMCPF severe necrotizing myositis with capillary pathology and fibrosis, TASS typical antisynthetase syndrome, TRI tubuloreticular inclusions, VAS visual analogue scale. * endplate zone. Scales: VAS ranges from 0-10 for severity of muscle damage where 0=no damage and 10=severe damage; all other changes are rated from "-" to "+++" where "-"=not present and "+++"=high presence.

Supplementary Table 6. Semiquantitative scoring system of patient and control cases based on large-scale electron microscopy datasets

	Histo	Analyzed capillaries	BM thickening (a)			BM reduplication (b)			Endothelial activation (c)			Ensheathment (d)			TRI (e)			AS	ACS a	ACS b	ACS c	ACS d
Patient 1	MMCP	100	0	50	50	75	25	0	92	8	0	70	30	0	100	0	0	2.13	1.50	0.25	0.08	0.30
Patient 2	MMCP	100	0	39	61	98	2	0	93	7	0	82	18	0	100	0	0	1.88	1.61	0.02	0.07	0.18
Patient 3	MMCP	100	0	3	97	16	74	10	41	39	20	31	57	12	100	0	0	4.51	1.97	0.94	0.79	0.81
Patient 4	MMCP	90	13	70	7	51	37	2	81	9	0	75	15	0	90	0	0	1.66	0.93	0.46	0.10	0.17
Patient 5	MMCP	100	0	30	70	72	22	6	85	14	1	85	9	6	100	0	0	2.41	1.70	0.34	0.16	0.21
Patient 6	MMCP	44	7	22	15	44	0	0	38	6	0	31	13	0	44	0	0	1.61	1.18	0.00	0.14	0.30
Patient 7	MMCP	100	5	50	45	62	34	4	64	34	2	71	28	1	100	0	0	2.5	1.40	0.42	0.38	0.30
Patient 8	MMCP	100	9	31	60	7	71	22	50	30	20	32	42	26	98	2	0	4.3	1.51	1.15	0.70	0.94
Patient 9	MMCP	100	46	52	2	62	38	0	72	26	2	95	5	0	100	0	0	1.29	0.56	0.38	0.30	0.05
Patient 10	MMCP	100	6	73	21	37	57	6	54	34	12	48	48	4	100	0	0	2.98	1.15	0.69	0.58	0.56
Patient 11	MMCP	100	14	80	6	99	1	0	89	11	0	92	7	1	100	0	0	1.13	0.92	0.01	0.11	0.09
Patient 12	MMCP	57	12	33	12	39	17	1	50	5	2	41	16	0	57	0	0	1.77	1.00	0.33	0.16	0.28
Patient 13	IVPP	100	11	65	24	28	72	0	40	49	11	52	43	5	96	4	0	3.09	1.13	0.72	0.71	0.53
Patient 14	IMNM+	100	0	64	36	44	56	0	90	9	1	67	29	4	100	0	0	2.4	1.36	0.56	0.11	0.37
Patient 15	IMNM+	77	2	33	42	2	66	9	34	34	9	23	43	11	76	0	1	4.13	1.52	1.09	0.68	0.84
Patient 16	IVPP	100	3	50	47	13	74	13	32	36	32	27	60	13	98	2	0	4.3	1.44	1.00	1.00	0.86
Patient 17	TASS	100	0	17	83	2	61	37	13	43	44	6	38	56	100	0	0	5.99	1.83	1.35	1.31	1.50
Patient 18	SNMCPF	67	0	13	54	5	52	10	13	18	36	17	30	20	67	0	0	5.27	1.81	1.07	1.34	1.04
Control 1	DM	59	36	22	1	53	6	0	14	25	20	30	26	3	27	8	24	2.15	0.41	0.10	1.10	0.54
Control 2	DM	44	26	14	4	42	2	0	19	10	15	37	6	1	37	4	3	1.63	0.50	0.05	0.91	0.18
Control 3	DM	100	10	58	32	50	50	0	67	29	4	60	33	7	61	21	18	2.56	1.22	0.50	0.37	0.47
Control 4	DM	100	70	23	7	85	15	0	69	26	5	69	28	3	58	11	31	1.22	0.37	0.15	0.36	0.34
Control 5	DM	100	13	86	1	100	0	0	95	3	2	98	2	0	92	3	5	0.97	0.88	0.00	0.07	0.02
Control 6	DM	100	0	89	11	98	2	0	98	2	0	92	7	1	100	0	0	1.24	1.11	0.02	0.02	0.09
Control 7	DM	80	1	73	6	70	10	0	79	1	0	58	20	2	57	5	18	1.5	1.06	0.13	0.01	0.30
Control 8	DM	100	93	7	0	95	5	0	96	4	0	83	14	3	64	16	20	0.36	0.07	0.05	0.04	0.20
Control 9	AS	100	7	58	35	62	38	0	62	35	3	56	39	5	95	4	1	2.56	1.28	0.38	0.41	0.49
Control 10	AS	100	3	50	47	16	80	4	27	38	35	19	47	34	100	0	0	4.55	1.44	0.88	1.08	1.15
Control 11	HC	100	97	3	0	97	3	0	99	1	0	99	1	0	100	0	0	0.08	0.03	0.03	0.01	0.01

Abbreviations: ACS average category sum score, AS antisynthetase syndrome, AS average sum score, BM basement membrane, DM dermatomyositis, HC healthy control, Histo histological diagnosis, IMNM+ immune-mediated necrotizing myopathy with MHC II positivity, IVPP inflammatory vasculopathy with perimysial pathology, MMCP minimal myositis with capillary pathology, SNMCPF severe necrotizing myositis with capillary pathology and fibrosis, TASS typical antisynthetase syndrome, TRI tubuloreticular inclusions. Capillaries were analyzed regarding BM thickening (category a), BM reduplication (category b), endothelial activation (category c), ensheathment (category d) and presence of TRI (category e). Here, each of the three columns per category shows the number of capillaries with the scores “0” (left), “1” (middle) and “2” (right).

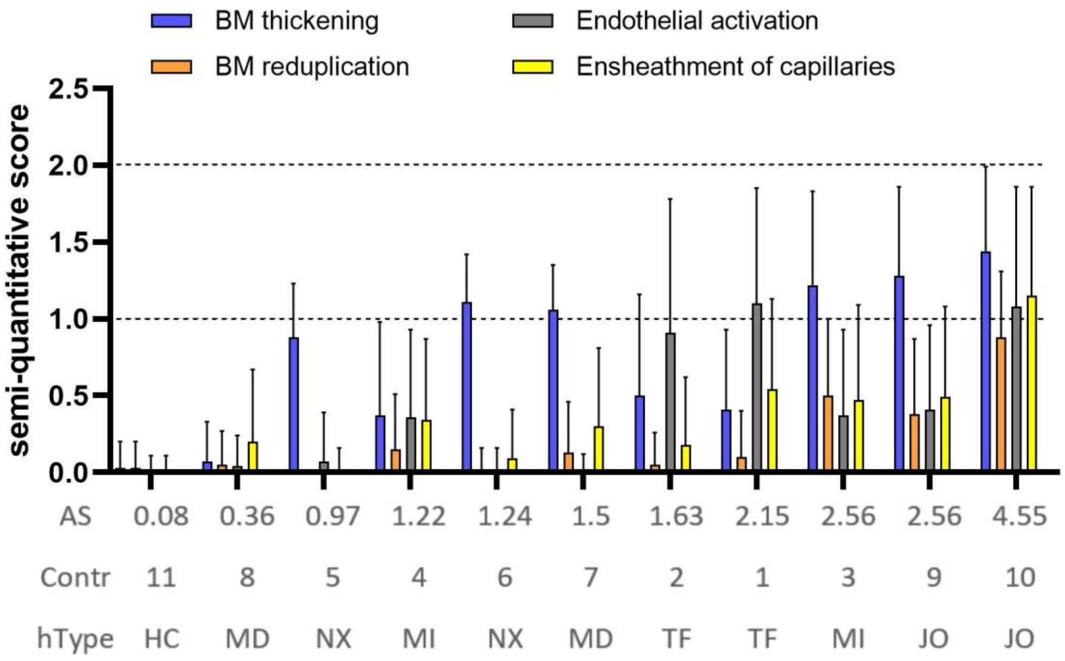

Supplementary Fig. 1 Representation of capillary scoring analysis of all control cases (Contr) based on large-scale digitization datasets. Average category sum scores of basement membrane (BM) thickening (blue), BM reduplication (orange), endothelial activation (gray) and ensheathment of capillaries by pericyte and endothelial processes (yellow). Note that the controls demonstrate very different alterations of these four categories as well as the average sum score (AV) depending on the histological pattern (hType) of the muscle biopsy. Note that controls demonstrate either normal or relatively normal (8,11) capillary ultrastructure, mild alterations that are mainly caused by BM thickening (5,6,7), alterations dominated by endothelial activation (1,2; both TF) and mild to pronounced alterations (3,4; MI and 9,10; JO). Abbreviations: Contr control case, HC healthy control, Jo Jo-1 associated antisynthetase syndrome, MD MDA-5 associated dermatomyositis, Mi Mi-2 associated dermatomyositis, NX NXP-2 associated dermatomyositis, TF TiF1-y associated dermatomyositis. Displayed are bar graphs with mean and SD.
